# Evaluation of Preclinical Efficacy of Curcumin-Loaded Bicosome Systems in Amelioration of Oral Mucositis

**DOI:** 10.3390/pharmaceutics17020181

**Published:** 2025-02-01

**Authors:** Daniela Vergara, Claudia Sanhueza, Susana Méndez, Mariela Bustamante, Benjamín Vega, Francisca Acevedo, Olga López

**Affiliations:** 1 Center of Excellence in Translational Medicine—Scientific Technological Bioresource Nucleus (CEMT-BIOREN), Faculty of Medicine, Universidad de La Frontera, Temuco 4811230, Chile; s.mendez03@ufromail.cl (S.M.); francisca.acevedo@ufrontera.cl (F.A.); 2 Laboratory of Pharmaceutical and Cosmetic Bioproducts, Center of Excellence in Translational Medicine (CEMT), Faculty of Medicine, Universidad de La Frontera, Temuco 4811230, Chile; 3 Center for Resilience, Adaptation and Mitigation (CReAM), Universidad Mayor, Temuco 4780000, Chile; claudia.sanhuezas@umayor.cl; 4 Escuela de Ingeniería, Facultad de Ciencias, Ingeniería y Tecnología, Universidad Mayor, Temuco 4780000, Chile; 5 Center of Food Biotechnology and Bioseparations, Scientific and Technological Bioresource Nucleus BIOREN, Universidad de La Frontera, Temuco 4811230, Chile; mariela.bustamante@ufrontera.cl; 6 Chemistry and Pharmacy Undergraduate Program Faculty of Medicine, Universidad de La Frontera, Temuco 4811230, Chile; b.vega05@ufromail.cl; 7 Department of Basic Sciences, Faculty of Medicine, Universidad de La Frontera, Temuco 4811230, Chile; 8 Department of Chemical and Surfactant Technology, Institute of Advanced Chemistry of Catalonia (IQAC-CSIC), C/Jordi Girona 18-26, 08034 Barcelona, Spain; olga.lopez@iqac.csic.es

**Keywords:** curcumin, bicosomes, delivery systems, ex vivo skin permeation, oral mucositis

## Abstract

Background/Objectives: Oral mucositis (OM) is a common and debilitating side effect of cancer therapy, characterized by ulceration or inflammation of the oral mucosa. This study evaluates the preclinical efficacy of curcumin-loaded bicosome systems (cur-BS) in mitigating chemotherapy-induced OM in mice. Methods: BS were prepared using a combination of 1,2-di-palmitoyl-sn-glycero-3-phosphocholine (DPPC) and 1,2-dihexanoyl-sn-glycero-3-phosphocholine (DHPC), α-tocopherol, and curcumin, encapsulated within liposomal vesicles. Three formulations with different curcumin concentrations (180, 540, and 900 μM) were characterized by particle size, polydispersity index (PDI), encapsulation efficiency (EE), appearance, and morphology. The formulation with the highest concentration (cur-BS 5×) was selected for ex vivo permeability studies, release profile analysis, and in vitro anti-inflammatory efficacy. OM was induced in mice using 5-fluorouracil (5-FU) and acetic acid. Cur-BS 5× was compared to the commercial product Dentoxol^®^. Results: The results showed that cur-BS 5× provided sustained release through a mechanism involving both diffusion and matrix relaxation, enhancing curcumin retention in deeper skin layers. Treatment with cur-BS 5× downregulated the expression of inflammatory markers (IL-1β and TNF-α). Macroscopic assessments demonstrated that both cur-BS 5× and Dentoxol^®^ reduced OM severity, with the greatest improvement observed between days 6 and 9. By day 24, OM scores were 1.25 ± 0.5 for cur-BS 5× and 1.0 ± 0.0 for Dentoxol^®^, indicating effectiveness in both treatments. However, histological analysis revealed superior tissue recovery with cur-BS 5×, showing better epithelial structure and reduced inflammation. Cur-BS 5×-treated mice also exhibited greater weight recovery and higher survival rates compared to the Dentoxol^®^ group. Conclusions: These findings suggest that cur-BS 5× may enhance OM treatment, offering outcomes comparable to or better than those of Dentoxol^®^.

## 1. Introduction

Oral mucositis (OM) is a common and debilitating complication of radio chemotherapy, posing a significant global public health challenge [1]. Affecting up to 40% of cancer patients undergoing chemotherapy, the incidence of OM rises to 80% among those receiving high-dose chemotherapy and approaches 100% in patients treated for head and neck cancers [2,3]. Annually, approximately 600,000 new cases of OM are reported worldwide [4]. Clinically, OM is characterized by the thinning of the oral epithelium, inflammation, ulceration, pain, bleeding, and an increased risk of secondary infections [5]. These symptoms contribute to chronic pain, malnutrition, and weakened immunity, severely impacting quality of life and often leading to treatment delays, increased healthcare costs, and reduced survival rates [3]. The severity of OM is further exacerbated by concurrent oral health issues, such as dental diseases and infections, which can compound its effects. Risk factors for OM include both modifiable factors, such as smoking, alcohol consumption, and stress, and non-modifiable factors, such as genetic predispositions [6,7,8,9].

The pathogenesis of OM involves a complex cascade of events triggered by chemotherapeutic agents. These agents induce oral mucosal injuries by directly damaging cells, increasing reactive oxygen species (ROS), and activating innate immune responses [10]. This process leads to the activation of transcription factors such as NF-κB, resulting in the upregulation of pro-inflammatory cytokines like IL-1β and stress responders like COX-2 [3]. This cascade ultimately causes connective tissue breakdown, basement membrane destruction, and ulcer formation [11]. Despite extensive research, an effective and standardized treatment for OM remains elusive [12]. The current standard of care primarily involves laser therapy, cryotherapy, and palliative interventions, including the application of local medications to manage pain and inflammation [3]. However, these treatments are often limited by the lack of targeted drugs, poor adhesion to the mucosal surface, and short drug contact duration, which reduces their overall effectiveness [13,14].

Recent advancements in local therapeutic options aim to provide more effective pain and inflammation control, striving for rapid and complete remission while minimizing the systemic side effects associated with broader treatment approaches [15]. The lack of robust preventive strategies has driven the pursuit of novel therapeutic targets, with recent studies emphasizing the critical role of managing oxidative stress in both the prevention and treatment of OM [16]. Radiotherapy, a common cancer treatment, has been shown to deplete plasma antioxidants and elevate levels of ROS, thereby weakening the body’s defenses against oxidative stress and intensifying treatment-related side effects. Growing evidence supports the therapeutic potential of antioxidants—such as epigallocatechin-3-O-gallate, ascorbic acid, tocopherols, polyphenols, and thiols—in mitigating the adverse effects of radio chemotherapy and improving patient outcomes [3,17].

Among natural polyphenolic compounds, curcumin, derived from turmeric, has shown promising therapeutic effects in reducing the incidence and severity of OM induced by chemotherapy and radiation [18,19,20]. Curcumin acts by suppressing pro-inflammatory cytokines, growth factors, and the expression of COX-2 and NF-κB [21]. Clinical studies, including pediatric trials using curcumin mouthwash, have demonstrated significant reductions in inflammation without adverse effects [22]. Meta-analyses suggest that mouthwashes containing curcumin, chamomile, honey, and benzydamine may offer protective benefits against OM [23]. Topical applications of curcumin have also been shown to accelerate wound healing in animal models [24]. However, challenges such as curcumin’s poor solubility, stability, and bioavailability limit its clinical effectiveness, which can potentially be overcome through nanotechnology-based delivery systems [25].

In this context, lipid-based nano-carriers, known as “bicosomes”, have emerged as promising delivery systems for fragile bioactive compounds like curcumin [26]. This innovative dual encapsulation technology involves embedding active ingredients within bicelles—disk-shaped nanostructures measuring 15–25 nm in diameter—which are further enclosed within the external lipid vesicles [26]. The unique structure of bicosomes, featuring an outer lipid membrane that protects both the bicelles and their cargo, provides enhanced protection and stability for bioactives, making them particularly well-suited for delivering both lipophilic and hydrophilic antioxidants to the oral cavity [27]. Bicosomes are stable across temperature variations, maintain a consistent size regardless of dilution, and are biocompatible due to their lipid-based composition [28], representing a highly effective transport system for curcumin in the treatment of OM.

Building on our previous work, we have conducted a detailed characterization of cur-bicosomes, examining their chemical composition, physical stability, appearance, and storage effects. To further this research, the aim of the present study was to evaluate the effect of curcumin-loaded bicosome systems (cur-BS) on chemotherapy-induced OM lesions in a murine model using 5-fluorouracil (5-FU), a chemotherapeutic agent that causes direct damage to the epithelial cells of the oral cavity. The study investigated the effects of different curcumin concentrations in three cur-BS formulations, assessing parameters such as particle size, polydispersity index (PDI), encapsulation efficiency (EE), appearance, and morphology. Additionally, cur-BS underwent comprehensive evaluation through in vitro release studies, in vitro anti-inflammatory efficacy, ex vivo permeation studies, and in vivo testing in a murine model of oral mucositis. The findings from this study could address the challenges associated with curcumin stability and penetration in the oral mucosa, providing a sustained release of antioxidant molecules and offering a promising new treatment strategy for OM.

## 2. Materials and Methods

### 2.1. Chemicals

1,2-di-palmitoyl-sn-glycero-3-phosphocholine (DPPC) and 1,2-dihexanoyl-sn-glycero-3-phosphocholine (DHPC) were purchased from Avanti Polar Lipids (Alabaster, AL, USA). Lipoid P-100 phosphatidylcholine (>97%) from non-(GMO) soybean was kindly supplied by Lipoid GmbH (Ludwigshafen, Germany). Cholesterol, curcumin (cur ≥ 65%), α-tocopherol (α-toc ≥ 96%), and Entellan^TM^ were purchased from Sigma-Aldrich (St. Louis, MO, USA). Chemotherapeutic agent 5-fluorouracil (5-FU) was obtained from Merck (Darmstadt, Germany). Purified water was obtained from an ultrapure water system (Thermo Scientific Barnstead MicroPure ST, Langenselbold, Germany). HPLC-grade chloroform, HPLC-grade acetonitrile, and HPLC-grade methanol were purchased from Merck.

### 2.2. Preparation of the Systems

#### 2.2.1. Bicelles

The bicellar formulation was prepared by the thin-layer dispersion method, following the methodology described in our previous study [26]. Briefly, bicelles were prepared using DPPC/DHPC at a 3.5:1 lipid molar ratio (*q*), α-tocopherol (600 μM), and curcumin at varying concentrations (180, 540, and 900 μM). The mixture was dissolved in chloroform into a round-bottom flask. The chloroform was removed with a rotary evaporator (Büchi Rotavapor R-100, Flawil, Switzerland) at 40 °C; thin lipid films were formed on the flask walls. The dried lipid films were hydrated with distilled water to reach 6% (*w*/*v*) of total lipid concentration. Bicellar formulations were prepared by subjecting the sample to several cycles of sonication and freezing until the sample became transparent. Curcumin-loaded bicelles were called cur-bicelles (three cur-bicelles were prepared: system 1×, 3×, and 5×).

#### 2.2.2. Bicosome Systems

The external lipid vesicles were prepared using Lipoid P-100/cholesterol in a ratio of 8:2 at 14% *w*/*v*. These two components were mixed in chloroform, and afterward a lipid film was formed by removing the chloroform by rotary evaporation at 40 °C. The film was hydrated with the cur-bicelles until bicosome systems (BS) formed, these BS were called cur-BS (three cur-BS were prepared: systems 1×, 3×, and 5×). Table 1 summarizes the components, concentrations, and percentage of mass (%) used for each formulation.

### 2.3. Determination of Particle Size and Polydispersity Index (PDI)

The particle size and PDI of the bicellar formulations and the cur-BS were determined using a Zetasizer Nano ZS (HT series, Malvern Instruments, Malvern, UK) at 25 °C. Conditions for measurement were defined according to Liu et al. [29]. The relative refractive index, i.e., the ratio of the refractive index of the phospholipids (1.490) to that of the dispersion medium (1.330) was 1.120. The absorption of the phospholipids was 0.001.

### 2.4. Determination of Encapsulation Efficiency (EE)

To determine the EE (%) of cur-BS (1×, 3×, and 5×), 50 μL of each system was centrifuged at 12,000× *g* for 30 min at 20 °C (Centurion Scientific Limited K2015R, Chichester, UK). The supernatant was discarded, and the precipitate was diluted in a mixture of acetonitrile, methanol, and water (88/8/4 *v*/*v*/*v*). The concentrations of curcumin were determined by a HPLC system Alliance Waters (Waters Inc., Milford, MA, USA) equipped with a pump (Waters, 2695), a column oven (NNNN), and a UV–vis photodiode array detector (Waters, 2996). The system was operated using the software Empower 3 (build 3471). The separation was carried out using a reversed phase C-18 Agilent Eclipse Plus (Agilent, Santa Clara, CA, USA) column (250 4.6 mm ID, 5 lm). The elution program comprised isocratic conditions with methanol/acetonitrile/water (88:8:4 *v*/*v*/*v*). The column was kept at 40 °C, and the mobile phase flow rate was 1 mL/min and detection wavelength was 425 nm. A sample of 20 μL was injected onto the column. The curcumin peak was obtained based on their retention time (8.6 min). Finally, curcumin concentration was calculated from standard curves obtained by reading the values of absorbance of solutions containing curcumin (0.5–75 ppm) with a R^2^ of 0.99. The *EE* was determined using the following Equation (1).

(1) $$EE\left(\%\right)=\frac{Loaded\;curcumin\;}{Initial\;amount\;curcumin}\times 100$$

### 2.5. Cryogenic Transmission Electron Microscopy (Cryo-TEM)

The preparation of cur-bicosome was visualized by the cryo-TEM method. A thin, aqueous film was formed by dipping and withdrawing a bare specimen grid from the suspension. Glow-discharged, holey carbon grids were used. After withdrawal from suspension, the grid was blotted against filter paper, leaving thin sample films spanning the grid holes. These films were vitrified by plunging the grid into ethane, which was kept at its melting point by liquid nitrogen using a Vitrobot (FEI, Eindhoven, The Netherlands), and by keeping the sample before freezing at 100% humidity. The thin films were vitrified at room temperature. The vitreous sample films were transferred to a Tecnai F20 microscope (FEI) using a cryotransfer (Gatan, Barcelona, Spain). Visualization was performed at 200 kV, at a temperature between −170 °C and −175 °C, under low-dose imaging conditions.

### 2.6. In Vitro Drug Release Profile

The in vitro drug release study was conducted to evaluate the release profile of the cur-BS 5×. The analysis utilized a Franz diffusion cell (Medicell International Ltd., Liverpool, London, UK) equipped with a dialysis membrane serving as the permeation barrier. The pre-activated dialysis membrane, with a molecular weight cut-off of 12–14 kDa, was positioned between the donor and receptor compartments of the Franz diffusion cell. The receptor compartment was filled with 3 mL of release medium, composed of absolute ethanol and distilled water in a 70:30 ratio, and allowed to equilibrate under stirring at 200 rpm and a controlled temperature of 37 °C. Subsequently, 200 μL of the cur-BS formulation was placed in the donor compartment atop the dialysis membrane. Samples (200 μL) were collected from the receptor chamber at specified time intervals (0.5, 1, 2, 4, 8, 10, 12, 24, 28, 32, 36, and 48 h), and an equal volume of fresh release medium (200 μL) was added back to maintain sink conditions throughout the experiment. The concentrations of curcumin were determined using HPLC, following the protocol outlined in Section 2.4.

### 2.7. Curcumin Release Kinetics

To understand the release mechanism, the percentage of cumulative curcumin released was fitted to various mathematical models, where three with the best fit (comparing the R^2^ values obtained) were chosen for analysis: Peppas–Sahlin, Higuchi, and Weibull. With the use of DDSolver^®^ dissolution kinetic modeling software [30], the data were fitted into each of the kinetic Equations (2)–(4).

(2) $$Peppas\text{–}Sahlin:\frac{M_{t}}{M_{\infty }}=K_{1}\;t^{m}+K_{2}\;t^{2m}$$

where *M_t_*/*M*_∞_ is the cumulative percentage of the released curcumin at time *t*, *K*_1_ is the Fickian kinetic constant, *K*_2_ is the erosion rate constant, and *m* is the Fickian diffusion exponent.

(3) $$Higuchi:\frac{M_{t}}{M_{\infty }}=K_{1}\;t^{\frac{1}{2}}$$

where *M_t_*/*M*_∞_ is the cumulative percentage of the released curcumin at time *t*, *K* is the rate constant of curcumin released.

(4) $$Weibull:log\;\left[-ln\left(1-m\right)\right]=blog\left(t-T_{i}\right)-log\;a$$

where *m* is the fraction of curcumin in solution at time *t*, *a* is the time scale of the process, *b* is the shape parameter, and *T_i_* is the lag time.

### 2.8. Ex Vivo Drug Permeation and Skin Retention

The ex vivo permeation study was conducted following the animal protocol approved by the Ethical Commission of Animal and Human Experimentation under the Spanish Government, with oversight from the Ethical Commission of the Autonomous University of Barcelona. The study aimed to evaluate the dermal permeation and retention of cur-BS across various tissues including ovine vaginal mucosa, porcine oral mucosa, and porcine skin. These samples were obtained from the Veterinary School of the Autonomous University of Barcelona (Spain). The tissue samples were mounted in a Franz diffusion cell with a 150 mm diameter. The cur-BS 5× was applied to the donor compartment, with assay conditions mirroring those used in the in vitro release studies. Samples (200 μL) were collected from the receptor chamber at predetermined intervals of 0.5, 1, 2, 3, 4, 5, 6, and 12 h. To maintain sink conditions, an equal volume (200 μL) of fresh media was replenished in the receptor chamber after each sampling. After 12 h, the tissue samples were removed from the Franz diffusion cell, thoroughly washed with PBS (pH 7.4), and then the tissue was divided using a scalpel into two parts: the superficial layers and the remaining tissue, both of which were then sectioned into small pieces. These parts were homogenized in 5 mL of ethanol, and the curcumin deposited within the tissue was extracted by centrifugation at 5000 rpm for 10 min at 25 °C. The permeated fluids were collected from the receptor compartments and analyzed. The curcumin content was subsequently determined using HPLC, following the protocol outlined in Section 2.4. The percentage of curcumin recovery from different fractions (surface layers, remaining tissue, and fluid permeated through the tissue) was calculated using Equation (5). The not detected curcumin percentage was calculated using Equation (6).

(5) $$Curcumin\;\left(\%\right)=\frac{C_{I}-\;C_{fraction\;analized}}{C_{I}}\times 100$$

where *C_I_* is initial concentration of curcumin in the cur-BS 5× formulation and *C_fraction analized_* corresponds to the surface layers, remaining tissue, or fluid permeated through the tissue, each calculated separately.

(6) $$Not\;detected\;curcumin\;\left(\%\right)=\frac{C_{I}-\;{(C}_{SL}+C_{R}+C_{P})\;}{C_{I}}\times 100$$

where *C_I_* is initial concentration of curcumin in the cur-BS 5× formulation, *C_SL_* is concentration of curcumin recovered from the superficial layer, *C_R_* is concentration of curcumin recovered from the remaining tissue, and *C_P_* is concentration of curcumin quantified in the permeate.

### 2.9. Cell Viability Assesment

A human THP-1 macrophage cell line was used to assess changes in the inflammatory response following exposure to cur-BS 5×. The control corresponded to THP-1 cells without cur-BS 5× treatment. Cells were cultured in 75 cm^2^ flasks with RPMI 1640 medium, supplemented with 10% fetal bovine serum (FBS, Fisher, Pittsburgh, PA, USA) and 1% penicillin-streptomycin, in accordance with ATCC guidelines. Cultures were maintained at 37 °C in a 5% CO_2_ atmosphere, with media changes performed when cell concentrations reached 8 × 10^5^ to 9 × 10^5^ cells/mL.

To assess cell viability, monocytes were first differentiated into macrophages using PMA. Specifically, 1 × 10^5^ cells were seeded per well in a 96-well plate and differentiated by adding PMA at a concentration of 100 ng/mL, followed by a 24 h incubation [31]. Macrophages were then stimulated with LPS (5 ng/mL) for 24 h to mimic an infection-like state. After stimulation, cells were exposed to varying concentrations of cur-BS 5× (1000, 100, 10, 1, 0.1 and 0.01 μM) for an additional 24 h. Cell viability was assessed using the MTT assay. Optical densities (OD) were measured at 570 nm using a microplate reader (BMG, LabTech, Offenburg, Germany). The percentage of cell viability was calculated using Equation (7).

(7) $$Cell\;viability\;\left(\%\right)=\frac{{OD}_{\left(sample\right)}-{OD}_{\left(blank\right)}}{{OD}_{\left(control\right)}-{OD}_{\left(blank\right)}}\times 100$$

where *OD*_(*sample*)_ refers to the absorbance of experimental groups treated with different curcumin concentration in BS, *OD*_(*control*)_ refers to the absorbance of the group containing only cells and medium, and *OD*_(*blank*)_ corresponds to the absorbance of pure medium.

#### qRT-PCR

To evaluate changes in TNF-α and IL-1β expression following exposure to cur-BS 5× systems, qRT-PCR analysis was performed. The assay was conducted in a 12-well plate using a concentration of 10 μM, identified as the minimum concentration that did not affect cell viability. Each well contained 1 × 10^6^ cells, and the protocol followed the method described by Adaileh et al. [32]. The control group included cells cultured exclusively in medium, without cur-BS 5×. Total RNA was extracted using Trizol (Invitrogen, Carlsbad, CA, USA), and miRNA-155 expression levels were measured by qRT-PCR using a 7500 Fast Real-Time PCR System with high-resolution melting software (Foster City, CA, USA). RNA concentrations were quantified with a Nanodrop 2000 spectrophotometer (Thermo Scientific, Waltham, MA, USA). One μg of total RNA was treated with DNase and subsequently converted into complementary DNA (cDNA) using All-in-One 5× RT MasterMix (ABM^®^) according to the manufacturer’s protocol. RT-PCR assays were performed using BRILLIANT II SYBR Green qPCR Master Mix (Agilent, USA) and the primer sets listed in Table 2. Relative transcription levels were calculated using the 2^−ΔΔCt^ method, with GAPDH serving as the reference gene for normalization.

### 2.10. Animal Models

Female BALB/c mice, 6–8 weeks old and weighing 15–23 g, were obtained from the Instituto de Salud Pública of Chile (ISP). The animals were housed in polycarbonate cages within a controlled environment, maintaining a 12 h light–dark cycle, humidity levels between 60 and 80%, and a temperature of 25 ± 2 °C. Water and pelleted food were provided ad libitum. Prior to the experiments, all mice were acclimated for 7 days. All experimental procedures were conducted in strict accordance with international protocols for laboratory animal care and use and were approved by the Ethics Committee on Animal Use of La Frontera University (approval number 051/21).

#### 2.10.1. Experimental Design

All animals were randomly divided into following 3 groups (*n* = 8/groups):

Group 1: Negative control, composed of mice that did not undergo the OM induction protocol.

Group 2: Positive control, where mice underwent the OM induction protocol and were treated with Dentoxol^®^ (Ingalfarma^®^, Bogotá, Colombia), a widely used commercial product for the management of complex ulcerative lesions in the oral mucosa. Dentoxol^®^ enhances cellular regeneration and turnover, aiding in the healing of oral wounds. Its composition includes xylitol, sodium bicarbonate, hydrogen peroxide, camphor, preservatives, flavoring agents, sweeteners, and water.

Group 3: Experimental group, where mice underwent the OM induction protocol and were treated with cur-bicosome formulation.

The animals received 20 μL per day of the saline solution, Dentoxol^®^, and cur-BS 5× for a total of 24 days. The body weight of the mice was measured every 24 to 48 h.

#### 2.10.2. OM Induction

**Figure 1:**
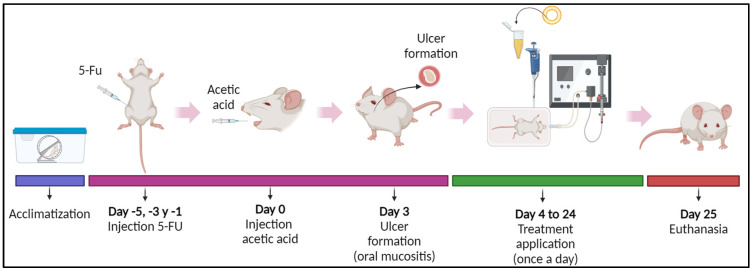
Schematic representation of the experimental design of oral mucositis (OM) induction by 5-FU.

To mimic the immunosuppression triggered by anticancer medications, mice received intraperitoneal injections of 5-FU on days -5, -3, and -1 of the experiment, at a dosage of 50 mg/kg body weight (*n* = 8 at each dose). Following this, on day 0, mucosal ulcers were induced by injecting 10% acetic acid (15 μL) using a microsyringe fitted with a 31-G needle (Figure 1) figure was cited. For the negative control group, both injections were administered using a 0.9% saline solution.

#### 2.10.3. Estimation of OM Severity Score

To evaluate the development and appearance of clinical signs of OM, once a day a macroscopic inspection of the animals’ oral cavities was performed under general anesthesia with isoflurane, following the scoring system reported by Sonis et al. [33] and Mohammed et al. [34]. OM scoring was defined as follows: 0 = normal; 1 = partial hyperemia, erythema, and swelling; 2 = overall hyperemia, erythema, and swelling; 3 = epidermolysis, hyperemia, and erythema; 4 = extensive epidermolysis and bleeding; 5 = bleeding and abscesses.

### 2.11. Histological Analysis

Upon finishing the experiments (day 25), all animals were euthanized. Samples of tongue and cheek obtained by biopsy were fixed in 10% buffered formaldehyde and stored in paraffin. Tissue sections (5 μm) were prepared, dewaxed, hydrated and stained with standard hematoxylin and eosin. The slices were then sealed with Entellan^TM^ and examined for pathological changes under an optical microscope (Euromex Oxion, Arnhem, The Netherlands). Images were captured using a Euromex VC.3036 camera (Euromex Oxion, Arnhem, The Netherlands).

### 2.12. Statistical Analysis

Statistical analyses were conducted using GraphPad Prism 9.0 software. Data from at least three independent experiments are presented as means ± standard deviations (SD). Statistical significance was evaluated based on one-way ANOVA followed by Tukey’s test and two-way ANOVA followed by Tukey’s test after validating the normality of the data set. A confidence level of α < 0.05 was considered statistically significant.

## 3. Results and Discussion

### 3.1. Characterization of Bicelles and Bicosomes

#### 3.1.1. Particle Size, Polydispersity Index (PDI), and Encapsulation Efficiency (EE)

The study examined the particle size of cur-bicelles and cur-BS using dynamic light scattering (DLS) (Table 3). Traditional particle size analysis based on scattered light intensity faces challenges with bicelles due to their small size, discoidal shape, and motion. In heterogeneous systems like bicosomes, particle contributions to scattered light intensity vary, possibly introducing measurement biases. Hence, this study analyzed results based on volume (%) to provide a more precise depiction of particle size [26,35].

**Figure 2:**
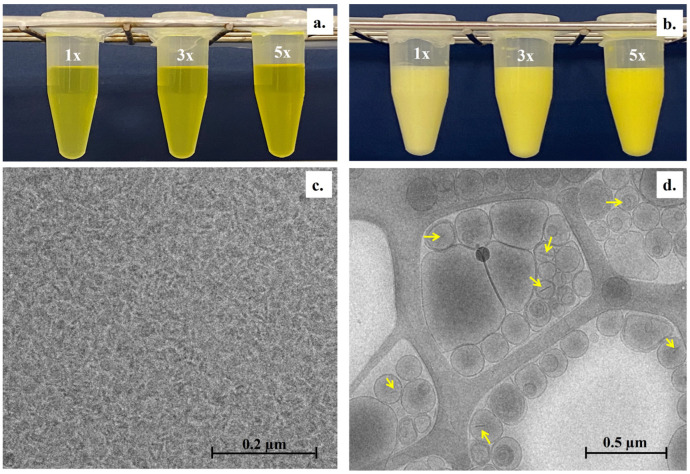
Macroscopic and microscopic characterizations of BS. (**a**) Cur-bicelles (systems 1×, 3×, and 5×), (**b**) cur-BS (systems 1×, 3×, and 5×), (**c**) cryo-TEM micrographs of cur-bicelles 5× sample, and (**d**) cryo-TEM micrographs of cur-BS 5× sample; the yellow arrow indicates the effective inclusion of cur-bicelles within the external lipid vesicle.

The particle sizes of cur-bicelles did not exhibit any significant differences (*p*-value < 0.05), measuring at approximately 15.1 ± 0.2 nm to 16.2 ± 0.2 nm. This indicates that the inclusion of different concentrations of curcumin did not affect the size of the bicelles. The PDI value, which represents the uniformity of particle size, is calculated as the ratio of the standard deviation to the mean particle size. A PDI value around 0.10 suggests monodispersity, values between 0.10 and 0.40 indicate a narrow particle size distribution, while values close to 1.0 suggest less uniformity in particle size [36,37]. For cur-bicelles, it was found to be 0.18 ± 0.01 to 0.34 ± 0.01, indicating a narrow particle size distribution. The visual aspect of the bicellar formulations can be observed in Figure 2a,b. The small average particle size and the homogeneity of cur-bicelles are advantageous characteristics for the stability, solubility, and bioavailability of the formulations [38].

On the other hand, the particle size of cur-BS presents a bi-modal size distribution or two main peaks (Table 4). Peak one exhibited particle sizes ranging from 376 ± 22 to 390 ± 2 nm, while peak two showed particle sizes ranging from 41 ± 1 to 52 ± 1 nm. The analysis by volume (%) revealed a higher proportion (≥60%) of small particles (≤60 nm) than large ones, indicating the predominant presence of small vesicles in the three systems. The bimodal distribution of the cur-BS increases the PDI until it reaches a value of 1.0 as expected. Histograms of particle size distribution (nm) by volume (%) for cur-bicelle (systems 1×, 3×, and 5×) and cur-BS (1×, 3×, and 5×) are shown in Figure S1.

For topical administration, lipid vesicles with a particle diameter ≥ 100 nm encounter difficulties in penetrating the deeper layers of the skin, remaining in the *stratum corneum*. However, these lipid vesicles can disassemble upon contact with dermal tissue, releasing their contents. In addition, particles with a diameter ≤ 50 nm are small enough to transport their cargo into the deeper layers of the skin, depositing it effectively in the dermis and epidermis [36,39]. In this context, our cur-BS, composed of curcumin-loaded bicelles encapsulated within an external lipid vesicle, offers a promising approach. This system can modify the skin’s barrier permeability, reinforce the lipid structure, and facilitate the targeted delivery of molecules, such as curcumin, to specific layers of the skin [40].

A key physicochemical parameter for optimizing cur-BS is the encapsulation efficiency (EE), which represents the fraction of curcumin encapsulated within the bicelles and the external lipid vesicle. High EE is essential for ensuring curcumin’s bioavailability. As shown in Table 4, cur-BS 5× exhibited the highest EE (70 ± 4.1%), followed by cur-BS 3× (56 ± 0.4%) and cur-BS 1× (36 ± 2.6%). The EE can be attributed to the hydrophobic nature of the phospholipids, which stabilize curcumin within their hydrophobic regions. In cur-BS, curcumin benefits from dual encapsulation opportunities: compounds not integrated into the phospholipid membrane of the bicelle can be encapsulated within the membrane of the external lipid vesicle. Particle size analysis revealed that cur-BS 5× presents a higher percentage of larger vesicles compared to the other systems, allowing it to accommodate a greater number of bicelles due to its larger internal and external diameters.

EE values in lipid systems are influenced by the encapsulating material, chemical properties, and processing conditions [41]. Additionally, the sensitivity of curcumin to environmental factors such as light and temperature may impact EE. The literature reports EE values for curcumin ranging from 40% to 95% [26,42,43], placing our findings within an acceptable range.

#### 3.1.2. Visual Appearance and Microscopic Structure

The visual analysis of the cur-bicelles indicated that they displayed a clear and transparent appearance, accompanied by a yellow color (Figure 2a). Conversely, cur-BS exhibited a milky yellow appearance, with the color intensity increasing with the rise in curcumin concentration, as expected (Figure 2b). Cryo-TEM images of the cur-bicelles and cur-BS were evaluated to confirm the morphology and inclusion of bicelles in the external lipid vesicle (Figure 2c,d). Cryo-TEM analysis revealed that the external lipid vesicle exhibited a spherical morphology being mainly unilamellar structures, although multilamellarity was also observed. The results (Figure 2c,d) confirmed a size between 50 and 400 nm and a spherical morphology, aligning with expectations from cur-BS.

Upon confirming that the curcumin concentration (180, 540, and 900 μM) does not affect the particle size or the stability of the BS and based on previous characterization and stability studies [26], subsequent analyses were conducted using the formulation with the highest curcumin concentration (cur-BS 5×).

### 3.2. In Vitro Curcumin Release Profile

**Figure 3:**
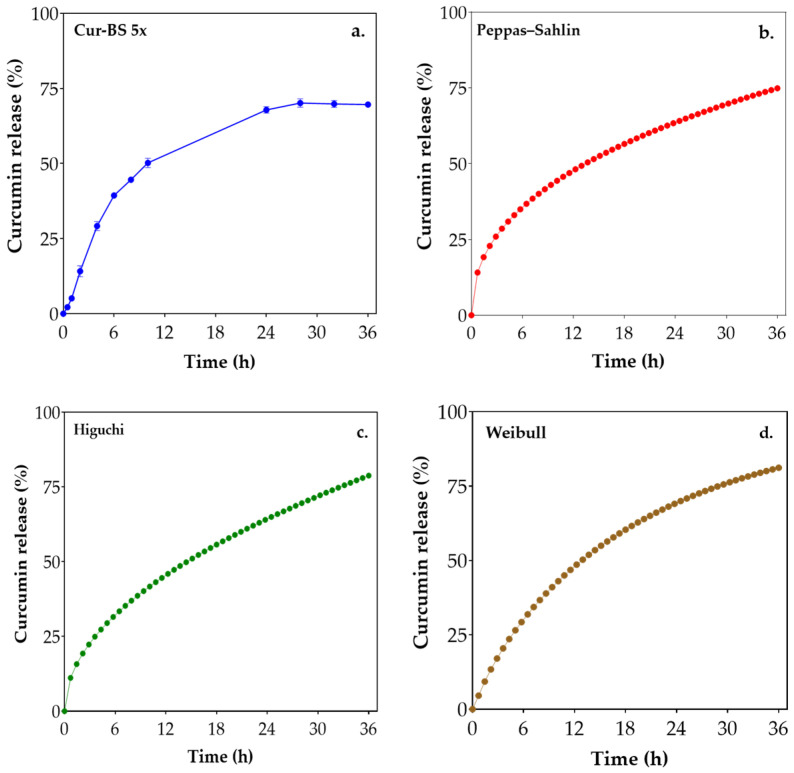
In vitro cumulative release profile of curcumin from cur-BS 5×. (**a**) Cumulative percentage of curcumin released in the time, (**b**–**d**) kinetic models for curcumin release, (**b**) Peppas—Sahlin model, (**c**) Higuchi model, and (**d**) Weibull model.

Mathematical models offer insights into drug release mechanisms and enable preliminary predictions of in vivo release profiles [44]. The cumulative release profile of the cur-BS 5× at 37 °C is illustrated in Figure 3. A rapid initial release of curcumin is observed within the first 12 h, during which approximately 50% of the curcumin is released into the receiving medium composed of absolute ethanol and distilled water (70:30 *v*/*v*). This initial burst release is likely driven by the diffusion of curcumin molecules located near the surface of the bicosomes, a common phenomenon in liposomal systems where the release mechanism depends on the hydrophilic/hydrophobic balance of the encapsulated compounds and the stability of the liposomal membranes [45].

The release kinetics were analyzed using three models: Peppas–Sahlin, Higuchi, and Weibull. As shown in Table 5, the Peppas–Sahlin model provided the best fit to the experimental data, with a regression coefficient (R^2^) of 0.95, followed closely by the Higuchi model (R^2^ = 0.94) and the Weibull model (R^2^ = 0.94). The parameters derived from the Peppas–Sahlin model, specifically *K*_1_ (16.66), *K*_2_ (−0.34), and *m* (0.45), suggest that curcumin release is predominantly governed by an anomalous transport mechanism involving both diffusion and matrix relaxation [46]. The *m* value of 0.45 indicates a release profile that is intermediate between pure Fickian diffusion (*m* = 0.5) and case II transport (*m* = 1), supporting the conclusion of a mixed release mechanism [47]. This behavior is typical of spherical systems like liposomes, where both the diffusion of the drug through the matrix and the relaxation or restructuring of the matrix contribute to the overall release process [47]. Additionally, the differences in the compositions of the lipid bilayers and the presence of dual effective diffusion barriers in the BS (bicelles and liposomes) may also contribute to the anomalous transport mechanism observed [48].

The Higuchi model’s release coefficient *K_h_* (13.13) suggests that diffusion is the primary mechanism controlling curcumin release, consistent with typical diffusion-driven processes where drug release is proportional to the square root of time [49]. The Weibull model, with parameters *α* (12.28), *β* (0.84), and *T_i_* (0.2), indicate an approximately exponential release, as the *β* value is close to 1, suggesting a matrix-type curcumin release [50].

These findings demonstrate that curcumin release from cur-BS 5× is a complex process, significantly influenced by both diffusion and matrix relaxation mechanisms. The initial burst release is likely due to the diffusion of curcumin near the BS surface, while sustained release is maintained by the relaxation of the BS matrix and the subsequent release of curcumin from the bilayer of the bicelles. This dual mechanism suggests that cur-BS 5× could serve as an effective drug delivery system for curcumin, potentially enhancing its bioavailability and therapeutic efficacy in topical applications.

### 3.3. Ex Vivo Curcumin Permeation Assays

**Figure 4:**
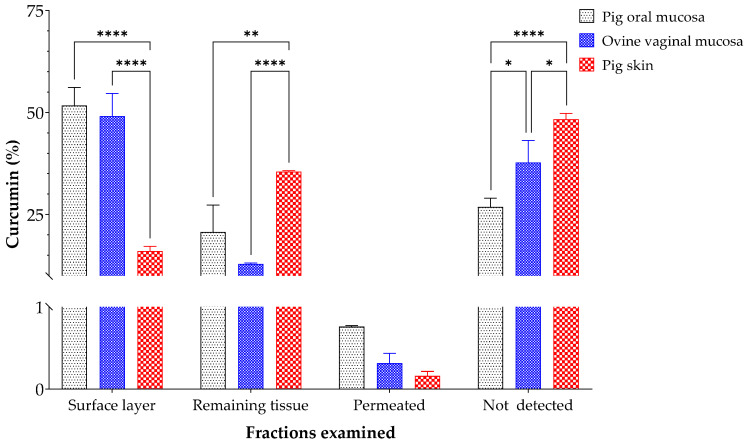
Ex vivo retention and penetration patterns of cur-BS 5× in three different tissues pig oral mucosa, ovine vaginal mucosa, and pig skin. Statistical analyses of two-way ANOVA, Tukey, and multiple comparisons: **** = *p* < 0.0001, ** = *p* < 0.01, and * = *p* < 0.05 (n = 3).

The ex vivo retention of curcumin from cur-BS was evaluated using Franz diffusion cells. Figure 4 illustrates the recovery of curcumin from three different tissues: pig oral mucosa, ovine vaginal mucosa, and pig skin. The analyzed fractions were categorized as curcumin retained in the surface layers (*stratum corneum* in the case of skin samples), curcumin in the remaining tissue, permeated through the tissue, and curcumin not detected. The results indicate that the percentage of curcumin recovered from the surface layer reaches 52 ± 4%, 49 ± 6%, and 16 ± 1% for pig oral mucosa, ovine vaginal mucosa, and pig skin, respectively. Meanwhile, the percentage of curcumin recovered from the remaining tissue is 21 ± 7%, 13 ± 0.2%, and 35 ± 0.3% for pig oral mucosa, ovine vaginal mucosa, and pig skin, respectively. The curcumin recovered from the receptor compartment of the Franz diffusion cells corresponds to the curcumin that permeated through the tissue, with very low values not exceeding 1% for the three types of tissues analyzed. Curcumin not found corresponds to the remaining percentage to complete the 100% initially used in the assays, with values of 27 ± 2% in pig oral mucosa, 38 ± 5% in ovine vaginal mucosa, and 48 ± 1% in pig skin. Curcumin not detected may have degraded during some steps of the percutaneous absorption procedure that involves a long period of temperature exposure and exhaustive extraction from the tissue and receiving fluid.

The results indicate that surface layer (*stratum corneum* for skin samples) is a significant barrier to the permeation of hydrophobic compounds like curcumin, which tend to accumulate in this lipid-rich layer rather than penetrating deeper skin layers [51]. The moderate penetration into the deeper layers of the oral mucosa, vaginal mucosa, and skin is influenced by the composition of the tissues, particularly their lipid and protein structures, which affect curcumin’s permeability. The dense lipid matrix and protein structure of the skin facilitate greater retention of curcumin compared to mucosal tissues [52]. Additionally, the presence of hair follicles, sebaceous glands, and sweat glands in pig skin may have contributed to greater retention of this compound through follicular and glandular penetration [53].

Studies conducted by Abla and Banga [54] using a mixture of polyphenols (catechins, resveratrol, and curcumin) administered through porcine ear skin established that 90% of the polyphenols were retained in the skin, and only 10% were quantified in the underlying skin, highlighting the difficulty in effectively administering curcumin through the skin. However, our cur-BS 5× significantly enhances curcumin retention, ensuring a higher concentration is delivered and retained in deeper layers of the tissue samples.

On the other hand, factors such as the physiological and pathological structure of the tissue, the interaction between the lipid system and the tissues, the deformability of vesicle-based lipid structures, the compression of vesicles through the physiological pores of the skin, as well as the size, charge of the particles, and lipid composition, significantly influence the penetration of delivery systems [48,51,55]. Additionally, the challenge of fully extracting curcumin may be linked to the various transport mechanisms utilized by BS as they move through the tissue, including intercellular, intracellular, and transfollicular pathways. During the analysis for curcumin recovery, it is possible that some of the curcumin encapsulated within bicosomes was transported via intercellular pathways, which may have hindered the complete removal of curcumin from the tissue [56].

### 3.4. In Vitro Anti-Inflammatory Efficacy

**Figure 5:**
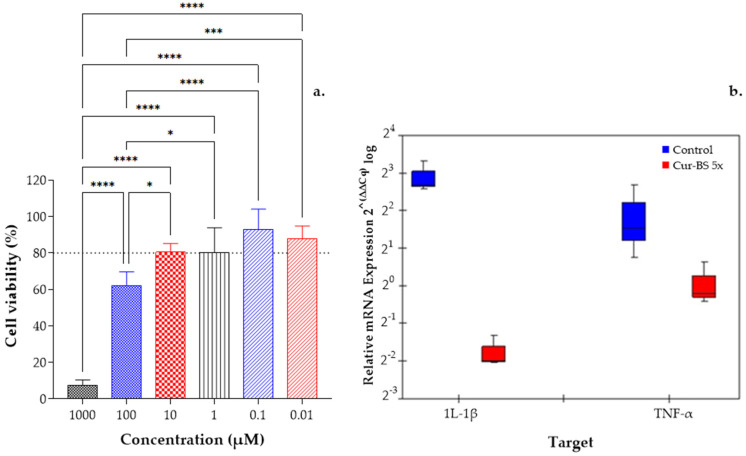
(**a**) Cytotoxic effects of cur-BS 5× on THP-1 cells. (**b**) In vitro anti-inflammation effects of cur-BS 5× LPS-induced THP-1 cells. The protein expression levels of IL-1β and TNF-α determined by qRT-PCR assay. Statistical analysis of one-way ANOVA, Tukey, and multiple comparisons: **** = *p* < 0.0001, *** = *p* < 0.001, and * = *p* < 0.1.

The cytotoxicity of cur-BS 5× was evaluated in THP-1 cells using the MTT assay. As shown in Figure 5a, cur-BS 5× exhibited dose-dependent cytotoxicity. At concentrations of 1000 and 100 μM, cell viability remained below 60%. However, at concentrations from 10 to 0.01 μM, cytotoxicity was greater than or equal to 80%, with no statistically significant differences observed within this range. Therefore, 10 μM was selected as the optimal concentration for in vitro anti-inflammatory assays.

Macrophage-secreted cytokines are essential for controlling inflammation. M1 macrophages produce pro-inflammatory cytokines, such as TNF-α and IL-1β, which initiate and sustain the inflammatory process. Conversely, M2 macrophages secrete cytokines like IL-4, IL-10, and VEGF, which help resolve inflammation and aid in tissue repair [57]. To evaluate whether cur-BS 5× can mitigate the inflammatory response in LPS-induced THP-1 cells, mRNA expression levels of IL-1β and TNF-α were quantified using qRT-PCR (Figure 5b). As expected, LPS stimulation upregulated the expression of these pro-inflammatory markers in the control group. However, treatment with cur-BS 5× effectively downregulated the gene expression and secretion of inflammatory factors, including IL-1β (*p* < 0.01) and TNF-α (*p* < 0.1). Similar effects were observed by Huang et al. [58], who also suggested that the superior anti-inflammatory effect of curcumin-loaded liposomes, compared to free curcumin, may be attributed to the sustained release properties of lipid-based drug delivery systems. When curcumin is retained in the lipid bilayer membrane, it can maintain its nanoform within lipid vesicles for an extended period, rather than existing in its molecular form in solution, thereby promoting macrophage uptake.

### 3.5. Assessment of Mucositis

OM is a debilitating disease characterized by inflammation and atrophy of the oral mucosa. The cur-BS 5×, a system where a wide variety of bilayers are present, seemed to favor this anti-inflammatory effect of curcumin. In this study, OM was induced in mice using intraperitoneal administration of 5-FU and acetic acid. The mice were divided into three groups: a negative control group with no mucositis induction, a positive control group treated with the commercial formulation Dentoxol^®^, and an experimental group treated with cur-BS 5×.

**Figure 6:**
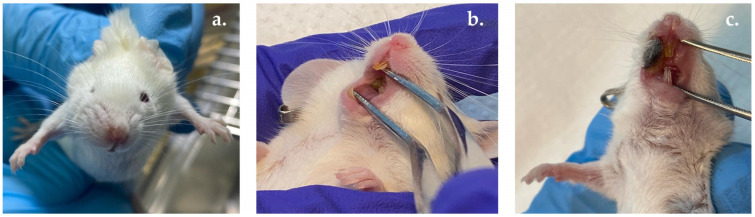
Preclinical appearance of buccal mucosa in mice following induction of oral mucositis (OM) using 5-fluorouracil (5-FU) and acetic acid: (**a**) inflammation; (**b**) ulceration; (**c**) necrosis.

Figure 6 illustrates the alterations observed in the murine model, clearly demonstrating the progression from severe inflammation to the development of ulcerations and necrosis in the oral mucosa. Specifically, Figure 6a highlights the early onset of inflammation, indicative of the cytotoxic effects of 5-FU on the oral epithelium. As the condition advances, subsequent images reveal deep ulcerations (Figure 6b) and necrotic tissue (Figure 6c), which are hallmark features of advanced mucositis. These visual findings underscore the destructive impact of 5-FU and acetic acid on the structural integrity of the oral mucosa, leading to significant tissue damage and lesion formation. This progression is consistent with the pathophysiological changes documented in previous studies on chemotherapy-induced mucositis, particularly when combined with the use of acetic acid [59,60].

**Figure 7:**
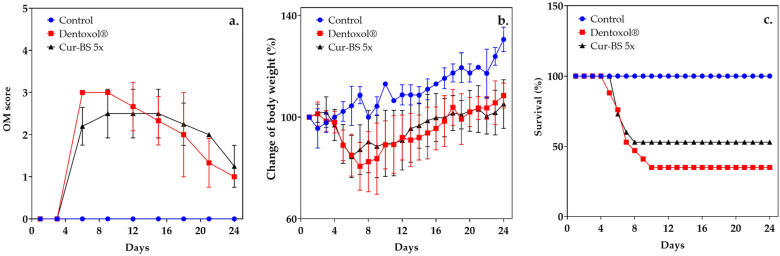
Effect of treatments in mice with oral mucositis (OM) induced by 5-fluorouracil (5-FU) and acetic acid. (**a**) Macroscopic scoring of the oral mucosa in the Dentoxol^®^ and cur-BS 5× treatment groups, with healthy animals serving as the control. (**b**) Variation in mice body weight over time. The percentage change in weight for each mouse was calculated relative to their body weight on day 0, which was set as the baseline at 100%. (**c**) Time course of survival analysis during treatments administration.

To evaluate the progression of OM, the oral cavities of the mice were examined, assessing ulcer formation and overall condition using a macroscopic scoring system (Figure 7a). The results showed that by day 3, OM severity had significantly increased in both the Dentoxol^®^ group and the cur-BS 5× group, reaching its peak between days 6 and 9. From day 18 onwards, the severity of OM gradually decreased in both groups. Although complete healing was not achieved by day 24, clear signs of recovery were observed. At the end of the study, the Dentoxol^®^ group recorded an OM score of 1.0 ± 0.0, while the cur-BS 5× group had a score of 1.25 ± 0.5, indicating positive effects from both treatments. However, the initial mucosal damage left lasting effects that prevented complete regeneration to a healthy state. These residual effects suggest permanent structural changes in the tissue, limiting full restoration of its normal function and appearance.

On the other hand, the body weight and survival rates of the mice were monitored over time, as shown in Figure 7b,c. The results demonstrated that the mice receiving the combination of 5-FU and acetic acid experienced significant weight loss and a marked decrease in survival compared to the healthy control group (Figure 7a,b). This chemotherapeutic agent is known to cause severe mucosal damage, significantly reducing nutritional intake due to oral pain and the presence of ulcers. This leads to pronounced weight loss. Additionally, the administration of 5-FU can induce morphological damage in the small intestine, resulting in diarrhea, which further contributes to the overall deterioration condition and eventually leads to increased mortality [34]. These findings are consistent with previous studies documenting the negative impact of 5-FU in preclinical models of OM, where the combination of chemical or mechanical irritation with chemotherapy exacerbates adverse effects on the mucosa [61].

However, after the initiation of treatment on day 7, both the group treated with Dentoxol^®^ and the group treated with cur-BS 5× began to regain lost weight, eventually returning to baseline levels. This recovery suggests that both treatments, the commercial one and our formulation, were effective in mitigating the weight loss associated with the induction of mucositis. Moreover, the group treated with cur-BS 5× showed a moderate weight loss and a significant improvement in survival rates compared to the group treated with Dentoxol^®^. This suggests that cur-BS 5× could be a viable alternative for protection against OM.

### 3.6. Histological Assays

**Figure 8:**
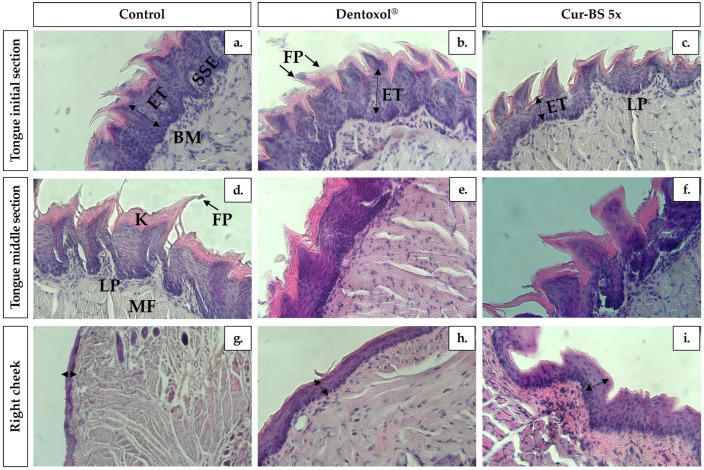
Histological analysis of oral tissue from mice subjected to oral mucositis induction with 5-FU and acetic acid, treated with Dentoxol^®^ and cur-BS 5×; the control group consists of healthy animals. (**a**–**c**) Initial section of the tongue, (**d**–**f**) middle section of the tongue, and (**g**–**i**) right cheek. Micrographs showing keratin layer (K), filiform papillae (FP), stratified squamous keratinized epithelium (SSE), basement membrane (BM), lamina propria (LP), and muscle fibers (MF), with epithelial thickness (ET). Tissues were stained with hematoxylin-eosin and observed under light microscopy at 20× or 40× magnification.

A histological analysis using hematoxylin-eosin staining of the tongue and cheek tissues was performed to evaluate the effects of Dentoxol^®^ and cur-BS 5× as post-treatment interventions on a 5-FU-induced OM model in mice. This analysis aimed to assess the structural changes in the tissues and determine the efficacy of these treatments in mitigating mucositis-related damage. The results obtained are shown in Figure 8.

In the initial and middle sections of the tongue for the control group (Figure 8a,d), the filiform papillae (FP) exhibit a normal structure with well-defined borders and intact epithelium covered by a keratin layer (K), showing no signs of inflammation or damage. In contrast, the Dentoxol^®^ treatment (Figure 8b,e) shows a more pronounced impact, with uneven thickening of the epithelial layers and areas of hyperkeratosis. The FP are less defined, suggesting an altered epithelial response, possibly due to abnormal or accelerated cell regeneration leading to compensatory thickening. In the cur-BS 5× treatment (Figure 8c,f), subtle morphological changes are noted, where the FP appear sharper and less prominent compared to the control, indicating possible mild epithelial atrophy. Although the epithelial thickness (ET) is reduced, there are no severe signs of inflammation or significant destruction of the epithelium. Studies conducted by Mohammed et al. [34] using a murine model of 5-FU-induced alimentary tract mucositis revealed that the 5-FU-treated group showed significant epithelial atrophy in the tongue, with reduced ET and destruction of FP. Severe ulcerative lesions, covered by necrotic material, were also observed, accompanied by inflammatory cell infiltration and active granulation, similar to the effects observed in this study.

In the right cheek, the control group epithelium (Figure 8g) maintains its normal structure, with no signs of atrophy or inflammatory infiltration. Dentoxol^®^ treatment (Figure 8h) induces ET, likely due to hyperplasia as a response to treatment-induced stimulation. Although the underlying connective tissue seems unaffected, the epithelium clearly responds to the stress induced by 5-FU. Finally, under the cur-BS 5× treatment (Figure 8i), there is a slight reduction in ET, which could suggest interference in cell renewal, though without significant inflammation. The relative effectiveness of cur-BS 5× can be attributed to two key factors. First, the unique structure of the BS system, composed of lipids organized into bilayers, which enhances the permeation and bioavailability of bioactive molecules. Second, the intrinsic therapeutic properties of curcumin play a pivotal role. Curcumin demonstrates anti-inflammatory, antioxidant, and tissue-regenerating abilities. It inhibits pro-inflammatory factors, reduces oxidative stress, and accelerates healing by promoting growth factors. Furthermore, curcumin protects epithelial cells by preventing premature cell death and exhibits antimicrobial properties, which collectively aid in reducing ulcer severity and effectively managing OM [62].

Finally, an important aspect to highlight is that α-tocopherol was incorporated in the formulation as a lipid membrane stabilizer owing to its excellent affinity for phospholipid fatty acids, improving the stability of membranes against adverse environmental factors such as oxidation and heat [63]. Similarly, α-tocopherol prevents oxidative damage [64], reduces cutaneous inflammation [65], promotes wound repair, and improves healing capacity [66].

These properties could generate a synergistic effect between curcumin and α-tocopherol present in cur-BS 5×. In particular, the combination of both compounds could enhance the antioxidant and anti-inflammatory activities of the preparation [26], responding to the joint capacity to neutralize reactive oxygen species, reinforce the structural stability of cell membranes, and modulate signaling pathways involved in inflammatory and oxidative stress processes. Such a synergistic effect would substantially contribute to the observed results, demonstrating the relevance of including α-tocopherol as an adjuvant in curcumin-based formulations.

## 4. Conclusions

To conclude the scientific article, the results of this study demonstrate that cur-BS offer a promising therapeutic approach for the treatment of chemotherapy-induced OM. Through both macroscopic and histological evaluations, cur-BS 5× exhibited comparable efficacy to the commercially available treatment Dentoxol^®^ in reducing OM severity. The formulation was particularly effective in promoting tissue recovery and reducing inflammation, as evidenced by enhanced epithelial structure and reduced histopathological damage in treated groups. Additionally, cur-BS 5× treated animals demonstrated superior weight recovery and survival outcomes compared to Dentoxol^®^. These findings highlight the potential of bicosome-based delivery systems for improving curcumin bioavailability and therapeutic effectiveness, making them a viable alternative for OM management in clinical settings. Future studies should focus on clinical trials to validate these preclinical results and explore the broader applicability of bicosome systems in treating other inflammation-related conditions.

## Figures and Tables

**Table 1:** Summary of the composition to prepare cur-BS 1×, 3×, and 5×.

Bicosome System	DHPC/DPPC Relación Molar (*q*)	Cur (μM)	α-Toc (μM)	Lipoid P-100/Chol Ratio	Total Bicelle Lipid Concentration (%*w*/*v*)	Total Liposome Lipid Concentration (%*w*/*v*)	Total BS Lipid Concentration (%*w*/*v*)
Cur-BS 1×	3.5:1	180	600	8:2	6	14	20
Cur-BS 3×		540					
Cur-BS 5×		900					

**Table 2:** Primer pairs used in the RT-PCR analyses.

Gene Target	Sequences	
	Forward 5′-3′	Reverse 5′-3′
GAPDH	TGGGGAAGGTGAAGGTCGGA	GGGATCTCGCTGCTGGAAGA
TNF-α	CTCTTCTGCCTGCTGCACTTTG	ATGGGCTACAGGCTTGTCACTC
IL-1β	CCACAGACCTTCCAGGAGAATG	GTGCAGTTCAGTGATCGTACAGG

**Table 3:** Particle size (nm), volume (%), and polydispersity index (PDI) of cur-bicelle (systems 1×, 3×, and 5×). Each value represents the mean ± standard deviation of at least three replicates. Different letters mean statistically significant differences with a *p*-value < 0.05.

Cur-Bicelle	Particle Size (nm)	Volume (%)	Polydispersity Index (PDI)
System 1×	15.2 ± 0.2 ^a^	99.8 ± 0.06 ^a^	0.22 ± 0.02 ^b^
System 3×	15.1 ± 0.1 ^a^	99.6 ± 0.06 ^b^	0.34 ± 0.01 ^a^
System 5×	16.2 ± 0.2 ^b^	99.9 ± 0.06 ^a^	0.18 ± 0.01 ^c^

**Table 4:** Particle size (nm) and volume (%) of two peaks for cur-BS 1×, 3×, and 5×, and encapsulation efficiency (EE) for cur-BS 1×, 3×, and 5×. Each value represents the mean ± standard deviation of at least three replicates. Different letters mean statistically significant differences with a *p*-value < 0.05.

Bicosome Systems	Peak 1		Peak 2		EE Cur (%)
	Particle Size (nm)	Volume (%)	Particle Size (nm)	Volume (%)	
Cur-BS 1×	383 ± 7 ^a^	40 ± 1 ^b^	41 ± 1 ^a^	60 ± 1 ^a^	36 ± 2.6 ^c^
Cur-BS 3×	376 ± 22 ^a^	42 ± 7 ^a^	46 ± 10 ^a^	58 ± 7 ^a^	56 ± 0.4 ^b^
Cur-BS 5×	390 ± 3 ^a^	52 ± 2 ^a^	52 ± 1 ^a^	48 ± 1 ^a^	70 ± 4.1 ^a^

**Table 5:** Kinetic parameters for curcumin release from cur-BS 5×.

	Peppas–Sahlin Model				Higuchi Model		Weibull Model			
	R^2^	*K*_1_	*K*_2_	*m*	R^2^	*K_h_*	R^2^	*α*	*β*	*Ti*
Cur-BS x5	0.95	16.66	−0.34	0.45	0.94	13.13	0.94	12.28	0.84	0.2

*K*_1_, *K*_2_, *m*, *K_h_*, *α*, *β*, and *Ti* correspond to kinetic parameters.

## Data Availability

Data are contained within the article and Supplementary Materials.

## Supplementary Materials

The following supporting information can be downloaded at: https://www.mdpi.com/article/10.3390/pharmaceutics17020181/s1, Figure S1: Histogram of the particle size distribution (nm). (**a**,**c**,**e**) cur-bicelles 1×, 3× and 5× by volume (%), (**b**,**d**,**f**) cur-BS 1×, 3× and 5× by volume (%).
