# Supplementary material for: Evaluation of Preclinical Efficacy of Curcumin-Loaded Bicosome Systems in Amelioration of Oral Mucositis
Source: Pharmaceutics. 2025 Feb 1;17(2):181. doi: 10.3390/pharmaceutics17020181 (PMC11860046; doi:10.3390/pharmaceutics17020181)
Supplement: Supplementary file 1 [file pharmaceutics-17-00181-s001.zip › pharmaceutics-3397105-supplementary.pdf]

Supplementary Material

Evaluation of preclinical efficacy of curcumin-loaded bicosome systems in amelioration of oral mucositis

Daniela Vergara [1,2*], Claudia Sanhueza [3,4], Susana Méndez [1], Mariela Bustamante [5], Benjamín Vega [6], Francisca Acevedo [1,7], Olga López [8]

[1] Center of Excellence in Translational Medicine—Scientific Technological Bioresource Nucleus (CEMT-BIOREN), Faculty of Medicine, Universidad de La Frontera, Temuco 4811230, Chile.
[2] Laboratory of Pharmaceutical and Cosmetic Bioproducts, Center of Excellence in Translational Medicine (CEMT), Faculty of Medicine, Universidad de La Frontera, Temuco 4811230, Chile
[3] Center for Resilience, Adaptation and Mitigation (CReAM), Universidad Mayor, Temuco, Chile.
[4] Escuela de Ingeniería, Facultad de Ciencias, Ingeniería y Tecnología, Universidad Mayor, Temuco, Chile.
[5] Center of Food Biotechnology and Bioseparations, Scientific and Technological Bioresource Nucleus BIOREN, Universidad de La Frontera, Temuco 4811230, Chile.
[6] Chemistry and Pharmacy Undergraduate Program Faculty of Medicine, Universidad de La Frontera, Temuco 4811230, Chile.
[7] Department of Basic Sciences, Faculty of Medicine, Universidad de La Frontera, Temuco 4811230, Chile.
[8] Department of Chemical and Surfactant Technology, Institute of Advanced Chemistry of Catalonia (IQAC-CSIC), C/Jordi Girona 18-26, 08034 Barcelona, Spain.

* Correspondence: daniela.vergara@ufrontera.cl (D.V)

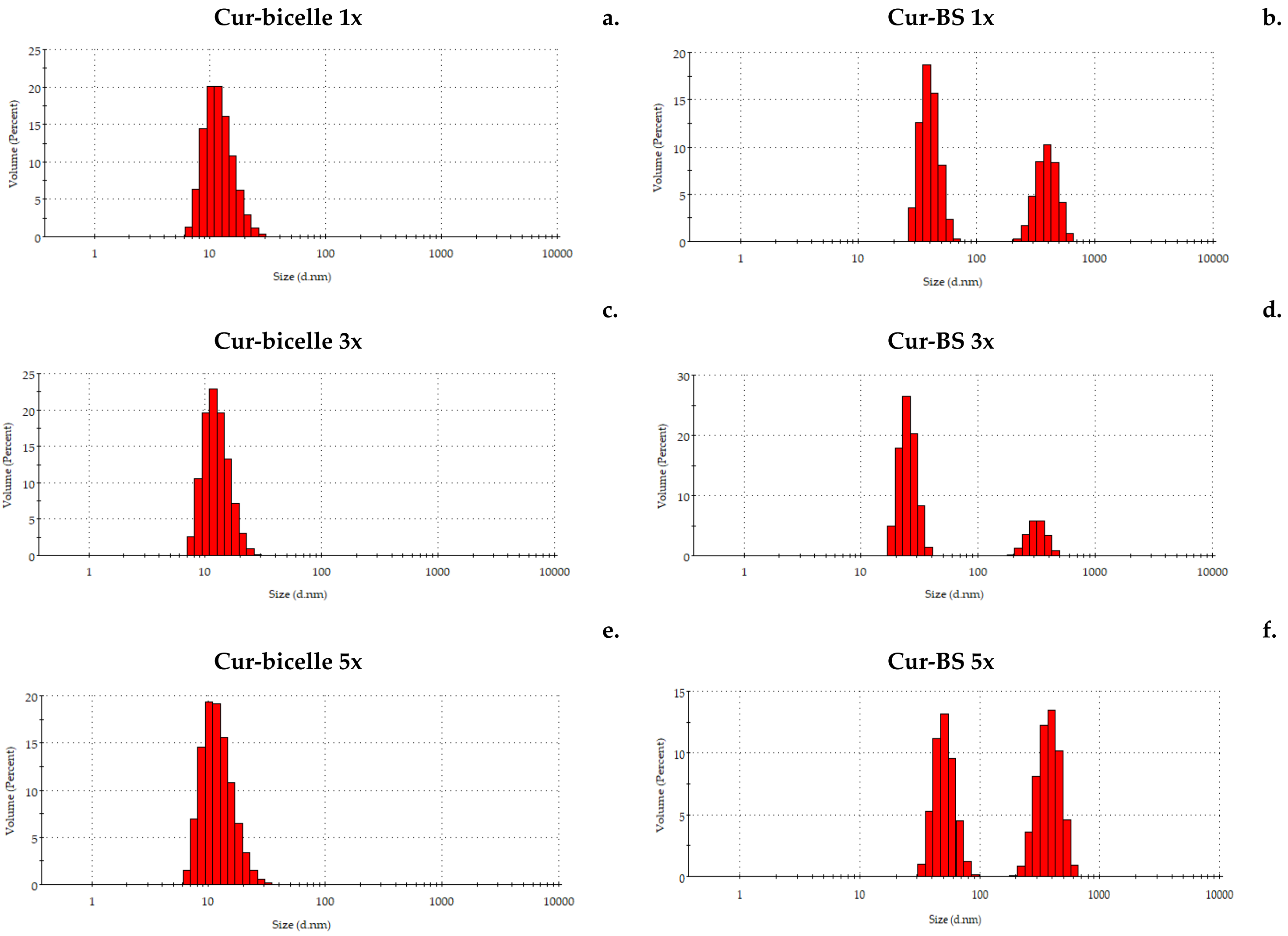

Figure S1. Histogram of the particle size distribution (nm). (a, c, e) cur-bicelles 1x, 3x and 5 x by volume (%), (b, d,f) cur-BS 1x, 3x and 5x by volume (%).
